# Probing behavior of *Adelges laricis* Vallot (Hemiptera: Adelgidae) on *Larix decidua* Mill: Description and analysis of EPG waveforms

**DOI:** 10.1371/journal.pone.0251663

**Published:** 2021-05-18

**Authors:** Katarzyna Dancewicz, Beata Gabryś, Iwona Morkunas, Sławomir Samardakiewicz

**Affiliations:** 1 Department of Botany and Ecology, University of Zielona Góra, Zielona Góra, Poland; 2 Department of Plant Physiology, Poznań University of Life Sciences, Poznań, Poland; 3 Laboratory of Electron and Confocal Microscopy, Faculty of Biology, Adam Mickiewicz University, Poznań, Poland; University of Saskatchewan College of Agriculture and Bioresources, CANADA

## Abstract

Adelgidae are a sister group of Aphididae and Phylloxeridae within Hemiptera, Aphidoidea and occur exclusively on Pinaceae. The piercing-sucking mouthparts of Adelgidae are similar to those of aphids and it is believed that adelgids ingest sap from both the non-vascular and vascular (phloem) tissues. The aim of the present study was to identify and characterize the adelgid stylet activities during their penetration in plant tissues. The probing behavior of *Adelges laricis* Vallot (Hemiptera: Adelgidae) on European larch *Larix decidua* Mill. and sucrose diets was monitored using the DC-EPG (Electrical Penetration Graph technique = electropenetrography). The EPG waveforms were described based on amplitude, frequency, voltage level, and electrical origin of the observed traces, and associated with putative behavioral activities based on analogy with aphid activities. Waveform frequency, duration, and sequence were analysed as well. *A*. *laricis* generated EPG signals at two clearly distinct voltage levels positive and negative, suggesting extracellular and intracellular stylet penetration, respectively. The adelgid EPG patterns were ascribed to four behavioral phases, which were non-probing, pathway, phloem, and xylem phases. Non-probing referred to the position of the stylets outside the plant tissues. Pathway phase was represented by three waveform patterns that visualized extracellular stylet penetration in non-vascular tissues without potential drops (AC1), with serial short (1.2–1.5 s) potential drops (AC2), and with ‘aphid-like’ (5–10 s) potential drops (AC3). Phloem phase comprised three waveform patterns at intracellular level, which in all probability represented phloem salivation (AE1), and phloem sap passive (AE2) and active ingestion (AE3). AE3 was a newly described waveform, previously unreported from Hemiptera. Waveform AG represented the ingestion of xylem sap. The comparative analysis demonstrated that the gymnosperm-associated adelgids show certain similarities in probing behavior typical of aphids and phylloxerids on angiosperm plants. The present work is the first detailed analysis of specific adelgid stylet activities on gymnosperms.

## Introduction

The conifer wooly aphids Adelgidae comprise 65 described species that occur exclusively on conifers and are highly host tree specific [1,2]. Adelgidae is a sister group of aphids Aphididae and phylloxerids Phylloxeridae within Aphidoidea (Hemiptera: Sternorrhyncha) [2–5]. Adelgidae and Phylloxeridae retained a number of evolutionary primary features as compared to Aphididae, therefore, some taxonomists prefer to classify these families as superfamiles [6–8]. In the present work, the Blackman and Eastop [3] approach is applied. Adelgidae and Phylloxeridae are distinguished from Aphididae by the absence of siphunculi, oviparity in all generations, and lack of the bacterial endosymbiont *Buchnera* [9–13]. While Phylloxeridae and the majority of Aphididae occur on angiosperms, Adelgidae occur exclusively on gymnosperms and only on Pinaceae. Several adelgid species are serious pests in coniferous forests in the Northern Hemisphere, specifically, *Adelges piceae* (Ratzeburg), *A*. *tsugae* Annand, *A*. *laricis* Vallot, *Pineus pini* Goeze, and *P*. *boerneri* Annand. The infestation of trees by adelgids may cause the production of abnormal wood, induction of galls, and limitation of branch development. Adelgid infestation of cones and needles can reduce seed yields, cause chlorosis and twisting or abscission of needles, hence reducing the tree vigour. Severe and chronic infestation may result in the loss of foliage, growth reduction, or mortality. Furthermore, adelgids produce copious wax and honeydew and if infestation is heavy, a mass of wax wool, honeydew and cast skins accumulates on the host tree [13–22].

Adelgids are cyclically parthenogenetic, entirely oviparous, and have complex, multigenerational, polymorphic life cycles that include an association with two species of host plants [13,23,24]. The primary host is always a species of the genus *Picea* A. Dietr. on which galls are induced, whereas the secondary host is one of the species of fir *Abies* Mill., larch *Larix* Mill., pine *Pinus* L., Douglas fir *Pseudotsuga* Carrière, or hemlock *Tsuga* Carrière, on which galls do not develop [12,13,25,26]. Adelgid generations on secondary host consist of wingless parthenogenetic oviparous morphs called exules [13]. The site on the secondary host is selected by the only mobile morph in a generation, the nymph of the first stage called the ‘crawler’, which hatches from the egg. The individuals of subsequent developmental stages (nymphs and adult females) remain in this spot for the rest of their lives, where they insert stylets, ingest sap, and lay eggs [12,13,22].

The adelgid piercing-sucking mouthparts forming the stylet bundle are similar to those of aphids. The stylet bundle is composed of four stylets, two of which (the mandibular stylets) fit together to form two canals. One canal, the salivary canal, injects saliva into plant tissues and the other, the food canal, transports nutrients from the tree to the insect [12,21]. However, adelgid stylets are much longer and more flexible than those of aphids, a trait that enables the insect to obtain access to the cells where the tree stores nutrients [22]. It is believed that adelgids insert their stylets and penetrate them deep within the plant tissue both intercellularly and intracellularly [12,16,21,27]. Adelgidae, similar to Aphididae, secrete a gelling saliva into the plant during probing (= stylet penetration) that builds up the salivary sheath around the path of the stylets and some cells are punctured and tapped along the stylet pathway [27].

Based on microscopy studies, it is understood that Adelgidae take their nourishment from various sources, the non-vascular (cortical parenchyma and xylem ray parenchyma) and vascular (phloem) tissue cells. Also each adelgid species presents a very specific probing behavior depending on the host plant, the developmental stage and type of ingestion cell [12,14,28,29]. *A*. *tsugae* ingests from xylem ray parenchyma cells of *Tsuga canadensis* L. [16,21], *Pineus pinifoliae* (Fitsh), whereas *P*. *strobi* (Hartig) take their nourishment from young sieve cells of the phloem of *Pinus strobus* L. [30,31], and *Adelges cooleyi* (Gillette) insert their stylets into the phloem cells when ingesting [32,33]. On the primary host, spruce, *Adelges abietis* L. and *A*. *laricis* ingest their food mainly from cortical parenchyma of the galls, which they penetrate intercellularly and intracellularly [27,28]. The galls induced by *A*. *abietis* and *A*. *laricis* form a strong nutrient sink with areas of nutritive tissue and links with vascular bundles of the stem, which ensures a continual supply of nutrients [20].

Very little is known about how *A*. *laricis* feeds on its secondary host plant, the European larch *Larix decidua* Mill. The existing knowledge on the probing and ingesting habits of Adelgidae is based on microscopy studies. Stylet activities during penetration have not been studied in Adelgidae in detail. Because stylet penetration of piercing-sucking phytophagous insects is hidden from the human eye, it is impossible to observe visually. The use of the electrical penetration graph technique also known as electropenetrography (both abbreviated EPG) is a good alternative to video recording [34–38]. In this experimental set-up, insect and plant are made part of an electrical circuit, which is completed when the insect inserts its stylets into the plant. Weak voltage is supplied to the plant and all voltage changes are recorded as EPG waveforms that have been correlated with stylet tip locations and insect-specific activities such as intra- or extracellular penetration, salivation, and ingestion [39]. This technique has been useful mainly in studies of aphid-plant interactions [40–48] but also in research on style penetration behavior of other sap-sucking insects, such as psyllids (Hemiptera: Psyllidae) [49,50], mealybugs (Hemiptera: Pseudococcidae) [51,52], thrips (Thysanoptera) [53,54], planthoppers (Hemiptera: Delphacidae) [55], leafhoppers (Hemiptera: Cicadellidae) [56–59], stink bugs (Hemiptera: Pentatomidae) [60] and phylloxerids [61,62]. EPG studies on Adelgidae were limited to *P*. *boerneri* on *Pinus taeda* L. and only two main waveforms, one extracellular and one intracellular were described [63]. Complete characterization and analysis of EPG signals of adelgids, such as *A*. *laricis*, and their correlation with specific probing behavioral activities have never been published.

The key objective of this research was to study the probing behavior of the larch wooly adelgid by identifying and characterizing the EPG waveforms generated by *A*. *laricis*,as well as explaining the behavioral meaning of these waveforms. This study will also provide future researchers with the methodology needed to study various aspects of adelgid–plant relationships, considering the complex life-cycle of these insects and the specificity of adelgid stylet penetration in plant tissues. This study also discusses the views on how the probing strategy of larch wooly adelgid relates to other phloem-feeding hemipterans.

## Materials and methods

### Insects and plants

**Fig 1:**
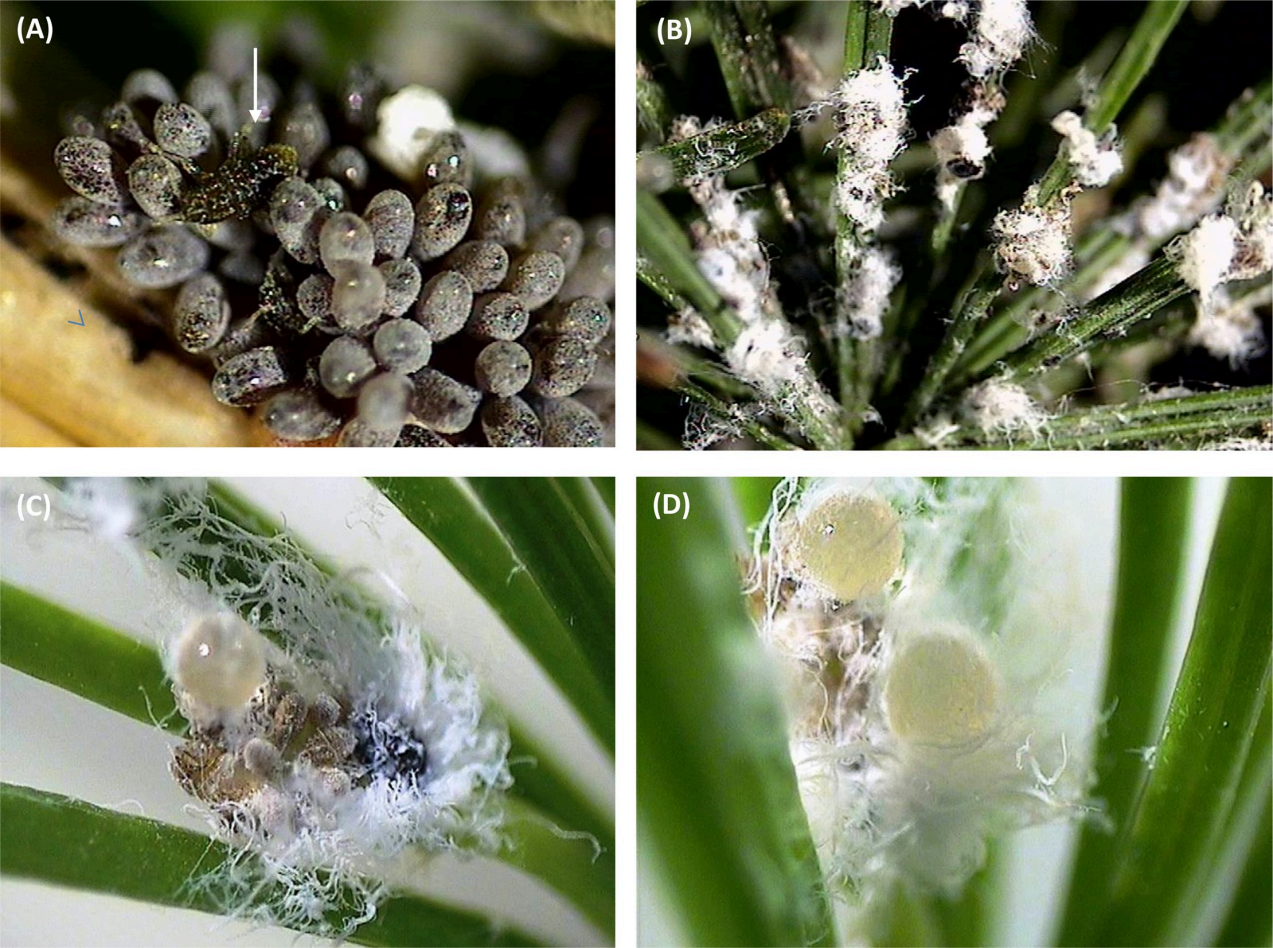
Parthenogenetic morphs of *Adelges laricis* exules on *Larix decidua*. (A) ‘Crawler’– 1^st^ instar nymph after hatching. The arrow points to the crawler. (B) ‘Wooly’ nymphs and females. (C-D) Wax-covered ‘spheres’ of accumulated honeydew. The ‘sphere’ is covered with wax and it often adheres to the insect and the egg clusters. Olympus DP-Soft binocular (10 x 6.3), Sony camera − Exwave HAD (1600 x 1200).

The individuals of the larch wooly adelgid, *Adelges laricis* Vallot, used in this study derived from two naturally infested larch trees, *Larix decidua* Mill. in Zielona Góra, Poland (one tree was located in private garden and the second tree was located at the building of Department of Botany and Ecology; the collection of material occurred at the consent of owners and administrators). The twigs with *A*. *laricis* exules (i.e., wingless, parthenogenetic morphs) and their eggs were cut from one- to two-year old larch shoots of mature trees using a pair of hand pruners. The twigs were collected from March to June when the needles on short shoots had completely developed. The twigs with *A*. *laricis* were transferred to the laboratory and kept under controlled conditions (20±1°C, 65±5% relative humidity, and 16:8 L:D photoperiod). In all experiments, crawlers (i.e., 1-2-day old nymphs that hatched from the eggs) (Fig 1A) and advanced, sessile nymphs and females were used (Fig 1B). The sessile morphs of *A*. *laricis* secrete wax, which forms a kind of a ‘wool’ to protect the body. Therefore, in the present study, the advanced nymphs and the females are called ‘wooly’ nymphs and ‘wooly’ females, respectively.

### Scanning electron microscopy (SEM)

Nymphs and adults of *A*. *laricis* parthenogenetic generations on *L*. *decidua* were observed with a scanning electron microscope Zeiss Evo 40 (SEM; Carl Zeiss SMT AG, Oberkochen, Germany) in the Laboratory of Electron and Confocal Microscopy, Faculty of Biology, Adam Mickiewicz University in Poznań, Poland. *A*. *laricis* remained on the larch needle during SEM preparation protocol. To obtain images of *A*. *laricis*, larch needle sections, previously colonized with *A*. *laricis*, were dehydrated in graded ethanol concentrations (60; 80; 90 and 95%) and, finally, in acetone, which was mixed with silica gel to withdraw water. Next, the samples were gold-sputtered and observed with a scanning electron microscope (SEM).

### Electrical penetration graph (EPG) recording

*Adelges laricis* probing behavior on needles of *L*. *decidua* and 20% sucrose diets was monitored using the Electrical Penetration Graph technique. Adelgids, for the majority of their lives are sessile, such that individuals of each developmental stage stay with their long stylets inside the host plant tissues. Consequently, EPG recording and analysis of results required a special approach. Two protocols were applied for EPG recordings, separately for the mobile crawlers and for the advanced, sessile ‘wooly’ nymphs and adult females. The crawlers, while walking, have their stylets withdrawn from plant tissues, so it was possible to apply the classical ‘aphid’ approach to the attachment of the EPG gold wire electrode. The crawlers were tethered to the golden wire electrode (1.5 cm long, 20 μm in diameter) and then the wired insects were individually placed on the needles of the larch and the EPG recording was started.

On the other hand sessile morphs never withdraw their stylets once they are inserted into plant tissues. Artificial removal of the insect from the plant causes the destruction of the stylet and results in the death of the insect [12]. Therefore, the sessile nymphs and adult females on needles were attached to a golden wire electrode while their stylets were already inserted into the plant. The gold wire (2 cm long, 20 μm diam.) was fixed with water-based conductive silver glue (EPG Systems, Wageningen, The Netherlands; www.epgsystems.eu) to the dorsum of the studied insect after some of the dorsal wax was delicately removed with a paintbrush to facilitate the connection to the gold wire.

For each recording, the quality of silver glue connection between the adelgid and the electrode was tested by using the EPG monitor’s calibration pulse after the first probe was initiated (crawlers) or during the probe (sessile morphs). A good contact was determined by an output signal in the form of a square pulse. The twigs of larch were cut short and put in beakers filled with water for the entire time of the EPG recording. The plant electrode was placed in the beaker. All experiments were carried out in a Faraday cage in the laboratory at 21±1°C, 65% of relative humidity, and 16:8 L:D photoperiod. The recordings were started at 9–10 a.m. and continued during the day for 8 hours without interruption. Each plant-adelgid set was considered a replication and was used only once. The number of replications was over 100. However, not all adelgids remained on the plant for the complete, 8-hour EPG experiment time. Therefore, during examination of the recordings, only the complete replications were kept for analysis, which were n = 84.

In addition to EPG recordings on larch needles, the probing behavior of crawlers of *A*. *laricis* was investigated using 20% sucrose diets. Pure 20% sucrose diet was chosen because it is highly phagostimulatory for aphids [64,65]. The experiment was conducted under controlled conditions at 20±1°C, 65±5% of relative humidity, and 16:8 L:D photoperiod. A total of 0.5 μl liquid diet was added to each ingestion chamber (3 mm diameter, 1 mm height), which was covered with one layer of stretched Parafilm M^®^ (sterilized with 75% ethanol), following the idea described by Sadeghi et al. [66]. Fresh diet was prepared just before the start of EPG recording. Early nymphs (crawlers) of *A*. *laricis* were collected from the stock colony and dorsally tethered on the abdomen with a gold wire (1.5 cm long, 20 μm in diameter) and water-based conductive silver glue (www.epgsystems.eu). After attachment of the wire electrode, the tethered adelgids were starved for 1 hr and after that period they were individually placed at the center of the surface of each diet chamber. The second electrode was introduced into the liquid diet. Probing behavior was monitored continuously for 4 hours with a four-channel DC EPG recording equipment. All experiments were started at 9–10 a.m.

Giga-4 and Giga-8 DC EPG systems with 1 GΩ of input resistance (EPG Systems, Dillenburg 12, 6703 CJ Wageningen, The Netherlands) were used to record the EPGs. The EPG signals were recorded and analyzed using Stylet+ Software (EPG Systems, Dillenburg 12, 6703 CJ Wageningen, The Netherlands). Signals were saved on the computer and the various behavioral phases were labelled manually using the Stylet+ software.

### Analysis of EPG recorded signals

The measurement criteria were similar to waveform characterizations used in aphid EPGs [37], which were relative amplitude, frequency, voltage level, and main electrical origin, either resistance (R) or electromotive force (emf) for each waveform. The relative amplitude of the waveform was the ratio between the measured amplitude (minimum to maximum deflection) and the plant voltage +5V. Waveform amplitude and frequency (Hz) were estimated basing on 30 randomly chosen events of each waveform (one to two events per recorded adelgid). The voltage level of the waveform, positive or negative, reflected an intracellular or extracellular position of the stylet tips, respectively [67]. The main electrical origin was due to either resistance fluctuation (R), electromotive force (emf), or both [36]. Electrical origin was investigated by using the normal or full EPG (formally called the DC system) containing components of resistance (R − electrical resistance changes caused by opening and closing of valves in the oral cavity, changes in concentration of electrolytes in the stylet canals, etc.) and electromotive force (emf − biopotentials such as membrane potentials of punctured plant cells or electrokinetic potentials caused by streaming fluids) origin [68,69]. In addition, the main electrical origin for each observed waveform was established by changing the plant voltage above, below, and on the 0 V level for applied signal to the food, as described for aphids (the best studied insects for EPGs) [36,68]. To determine the electrical origin, adjustments of applied signal voltages to positive and negative levels were performed in different periods for each observed waveform during the same recording (3–5 changes of the plant voltage per waveform).

The stylet activities and position of the stylet tips in the plant tissues were inferred from comparisons of the obtained traces to the published EPG-recorded waveform patterns of aphids and other Sternorrhyncha. Generally speaking, sternorrhynchan EPG waveforms have been found to be remarkably stereotypical [35–37,50,51,70,71]. The description and interpretation of all waveforms were based on the signals obtained from the DC EPG system applied in the present study. Nevertheless, it must be kept in mind that the use of alternative recording systems (AC-DC EPG systems) could reveal other morphological features of individual waveforms [72]. The use of honeydew clock was considered to monitor the sap ingestion-honeydew excretion activities of the studied individuals [73,74]. However, it appeared impossible due to the particular way the adelgids manage their honeydew. Honeydew excreted by adelgids does not form individual droplets but it is collected in the form of little wax-covered ‘spheres’ that remain entangled in the ‘wool’, close to the insect and the egg clusters (Fig 1C and 1D).

EPG waveforms were classified and named, by preceding a number with A for Adelgidae, similar to the ‘P’ proposed by Civolani et al. [50] for Psyllidae. Afterwards, the waveform frequency, duration, and sequence were analyzed. The following variables were calculated: number and proportion (%) of individuals showing a waveform, total waveform duration per individual, mean duration of an individual waveform event, and number of waveform events per individual. Additionally, the cell-puncturing adelgid activity was evaluated: number of individuals with potential drops during pathway phase, number of potential drops per individual, mean duration of individual potential drop, number of potential drops per 1 minute of pathway, and proportion (%) of different types of potential drops in the total number of potential drops.

The proportion (%) of different waveform durations in total probing time of *A*. *laricis* during 8-hour EPG experiment (all recordings) and the proportion of different types of individual waveform categories duration were also calculated: AC1, AC2, and AC3 in relation to complete AC phase (AC = AC1 + AC2 + AC3; n = 43) and AE1, AE2, and AE3 in relation to complete AE phase (AE = AE1 + AE2 + AE3; n = 77).

The sequential changes in adelgid behavior during probing were analysed as the proportion of time in each waveform at the end of each hour of the 8-h recording period basing on all recordings (n = 84). The evaluation was specified by also analysing selectively the recordings that started with the waveforms AC and AG (n = 24), or AE (n = 57). Those were the recordings of the sessile morphs that had their stylets inserted into plant tissues at the time of the start of the experiment, as described earlier.

The transitions from a certain waveform type to any other waveform type were calculated on the basis of the total 672 h of EPG recording (8 h x 84 individuals). All possible transitions from one waveform to the following one were analyzed [57]. The transition periods between two different waveforms (e.g., AC1-AC2, AE1-AE2, or AE2-AE3) were split in half between the two adjacent waveforms.

EPG variables describing adelgid stylet penetration activities were calculated manually and individually for every adelgid individual and the mean and standard errors were subsequently calculated using StatSoft, Inc. (2011) STATISTICA (data analysis software system), version 9.0. www.statsoft.com.

## Results

### EPG waveforms of *Adelges laricis* on *Larix decidua*

The EPG waveforms generated by larch wooly adelgids during stylet penetration in European larch needles were classified according to the waveform morphology (amplitude, frequency, shape) and electrical characteristics (voltage level, electrical origin). Based on comparative analysis, the EPG waveform patterns were ascribed to four major behavioral phases that have previously been distinguished in aphid stylet penetration activities on plants, which were non-probing (Np), pathway (C), phloem (E), and xylem (G) phases. Consequently, the probing phases revealed by *A*. *laricis* were labelled AC, AE, and AG, respectively, where ‘A’ stands for ‘*Adelges*’. The AC and AE phases were divided into variants AC1, AC2, and AC3 and AE1, AE2, and AE3, respectively and consistently with the specificity of their internal characteristics (Table 1).

**Table 1:** Characteristics and suggested meaning of EPG-recorded waveforms generated by *Adelges laricis* during probing on *Larix decidua*.

EPG waveform& sub-waveform labels	Relative amplitude (%)^a^ (min-max)	Frequency (Hz)	Voltage level^b^	Electrical origin^c^	Duration	Remarks	Possible meaning^d^
**AC**							
**AC1**	8 (4−14)	mixed/ irregular/ no pds	e	R	ca.1h	first waveform in the probe	pathway without intracellular punctures (any tissue)?
**AC2**	5 (2−14)	mixed/ irregular/ short pds	e/i	R/emf	ca.1 h	pathway with short and frequent ‘pd’s	pathway with intracellular punctures (any tissue)?
**AC3**	9 (2−30)	mixed/ irregular	e/i	R/emf	ca.1 h	pathway with ‘aphid-like’ and short‘pd’s	pathway with intracellular punctures within vascular tissue?
**Apd**							
**Apd1**	-	n. a.	i	R/emf	1.5−2.5 s	short and frequent ‘pd’s	intracellular punctures (any tissue)?
**Apd2**	-	n. a.	i	emf	5−10 s	‘aphid-like’ ‘pd’s	intracellular punctures within vascular tissue?
**AE**							
**AE1**	9 (4−20)	1−4/s	i	emf	sec−1 min	usually the first ‘E’, or between AE	salivation into sieve elements?
**AE2**	w: 8 (4−12)	w:4−5/s	i	R/emf	min−few hrs.	usually follows AE1	passive ingestion of phloem sap?
	p: 17 (10−26)	p: 0.5−1/s	i				
**AE3**	18 (10−32)	2−7/s	i	R/emf	min−few hrs.	usually follows AE2	active ingestion of phloem sap?
**AG**							
	20 (8−32)	0.5−2/s	e	emf/R	min−few hrs.	occurs between periods of AC waveforms	active ingestion of xylem sap?

AC–pathway phase, AC1, AC2, AC3 –variants of pathway phase; AE–phloem phase, AE1, AE3, AE3 –variants of phloem phase; Apd–potential drop, Apd1, Apd2 –variants of potential drops. AG–xylem phase; w–wave; p–peak; n.a.–not applicable.

^a^Medium amplitude; 5V = 100% amplitude.

^b^e–extracellular; i–intracellular.

^c^emf–electromotive force; R–resistance.

^d^Based on comparison with published studies on Aphididae.

#### Pathway phase: Waveforms AC

**Fig 2:**
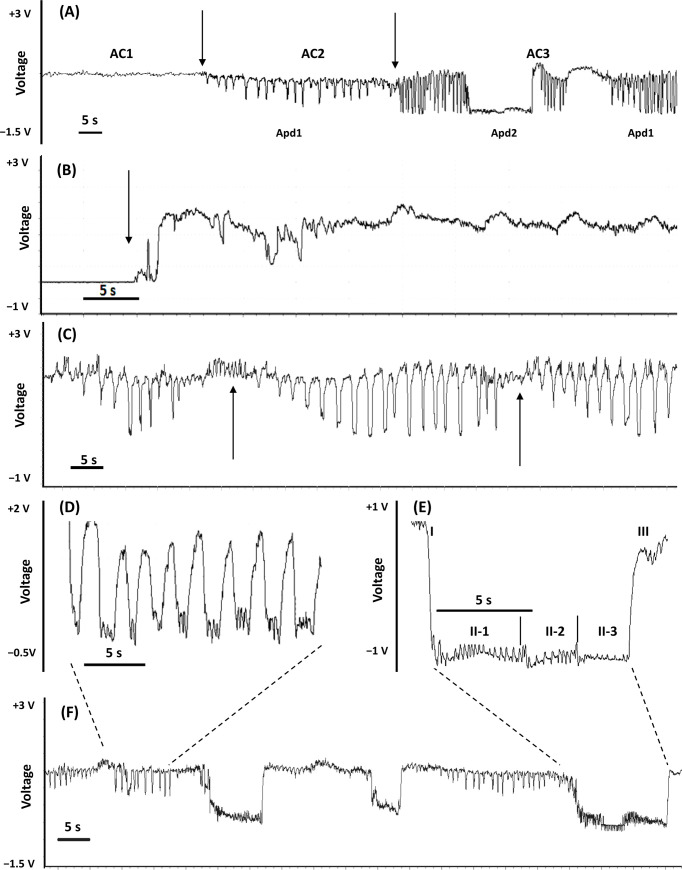
Pathway phase ‘AC’ waveforms generated by *Adelges laricis* during EPG-recorded probing on *Larix decidua*. (A) Compressed view of AC waveforms; arrows show the transition points; 100 s trace 2.5V resolution. (B) Non-probing and the transition to waveform AC1(arrow); 60 s trace, 2.5V resolution. (C) Waveform AC2; arrows indicate the beginning and the end of ‘short Apd1’ series; 100 s trace, 2.5V resolution. (D) ‘Short’ potential drops Apd1 –detail; 30 s trace, 2.5V resolution. (E) ‘Standard aphid-like’ potential drop Apd2; phases of Apd2 − I, II, III; sub-phases of Apd2 − II-1, II-2, II-3; 30 s trace, 2.5V resolution. (F) Waveform AC3, 100 s trace, 2.5V resolution.

Three variants of AC waveforms were distinguished: AC1, AC2, and AC3, consistently with the specificity of their internal characteristics. The baseline of all AC waveforms in the EPG recordings of *A*. *laricis* on larch needles occurred at positive voltage levels, but short events of potential drops of various durations also occurred in AC2 and AC3 (Fig 2A–2F).

**Fig 3:**
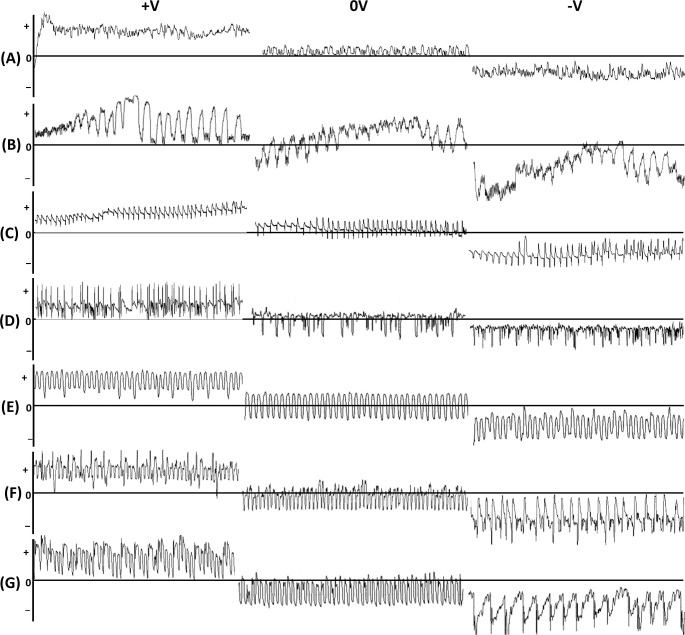
Assessment of the R and emf components contribution to each waveform by plant voltage adjustments, positive (+), zero (0) and negative (−). Adjustments of the plant voltage during recording. (A) Waveform AC1; 30 s traces, resolution 2.5V. (B) Waveform AC2; 60 s traces, resolution 2.5V. (C) Waveform AE1; 60 s traces, resolution 1V. (D) Waveform AE2; 60 s traces, resolution 1V. (E) Waveform AE3; 10 s traces, resolution 1V. (F) Waveform AE3; 30 s traces, resolution 1V. (G) Waveform AG; 30 s traces, resolution 1V.

Waveform AC1 occurred exclusively at positive voltage level and was composed of low amplitude waves of irregular frequency. Potential drops did not occur during AC1 (Table 1, Fig 3A). Adjustments of the input voltage demonstrated that the electrical origin of AC1 was mainly resistance (R) component because the amplitude and sign of this waveform pattern changed during the voltage adjustment (Table 1, Fig 3A).

The duration of AC1 per event was very variable, from few minutes to several hours. Individual AC1 events averaged per insect were about 1,5 h long and occurred almost 6 times during the 8-hour recording (Table 2). AC1 was detected in 76.7% of individuals of *A*. *laricis* with AC phase and it constituted 47.5% of total AC time.

**Table 2:** Frequency and duration of individual EPG-recorded waveforms generated by *Adelges laricis* during probing on *Larix decidua*.

EPG waveform	Number of recordings used for calculations	Number of individuals generating the waveform (%)	Total waveform duration per individual (min) Mean±SE (Min−Max)	Mean duration of individual waveform event (min) Mean±SE (Min−Max)	Number of waveform events per individual Mean±SE (Min−Max)
**Np**	n = 84	5/84 (5.6%)	125.9 ± 69.5 (18.5−378.6)	122.7 ± 70.2 (7.9−378.6)	1.4 ± 0.4 (1.0−3.0)
**AC**^**a**^	n = 43	43/84 (51.2%)			
**AC1**	n = 43	33/84 (39.3%)	100.0±23.2 (2.1−475.5)	46.0±17.2 (1.6−475.5)	5.7±1.4 (1.0−34.0)
**AC2**	n = 43	28/84 (33.3%)	110.4±22.5 (3.5−439.0)	23.6±6.1 (2.3−146.3)	8.4±1.6 (1.0−30.0)
**AC3**	n = 43	11/84 (13.1%)	50.7±16.2 (5.4−190.0)	15.1±3.6 (4.7−44.2)	3.8±1.1 (1.0−13.0)
**AE**^**b**^	n = 77	77/84 (91.7%)			
**AE1**	n = 77	53/84 (63.1%)	6.7±1.3 (0.2−44.6)	0.6±0.1 (0.1−2.8)	16.5±3.5 (1.0−137.0)
**AE2**	n = 77	73/84 (86.9%)	315.8±21.7 (0.8−480.0)	168.9±23.7 (0.4−480.0)	13.8±2.6 (1.0−134.0)
**AE3**	n = 77	31/84 (36.9%)	216.2±30.7 (6.4−480.0)	91.5±22.7 (2.1−480.0)	5.6±0.9 (1.0−19.0)
**AG**	n = 21	21/84 (25.0%)	77.2±28.0 (1.1−417.6)	50.6±16.6 (1.1−292.5)	4.8±1.5 (1.0−30.0)

Np–non probing, AC1, AC2, AC3 –variants of pathway phase, AE1, AE2, AE3 –variants of phloem phase, AG–xylem phase. n = number of replications (number of EPG-recorded individuals of *A*. *laricis*) used for calculations: number of all EPG recordings– 84; number of EPG recordings with AC phase– 43; number of EPG recordings with AE phase– 77; number of EPG recordings with AG phase– 21. Values are means±SE. Lowest and highest values in brackets (Min−Max).

AC^a^–total pathway phase (n = 43).

AE^b^–total phloem phase (n = 77).

Waveform AC2 was a complex pattern composed of activities at positive voltage levels and frequent short potential drops to negative level (Apd1) (Fig 2A, 2C and 2D). The component of AC2 at positive voltage level consisted of waves of very low amplitude and irregular frequency. The electrical origin of these sections of AC2 was composed primarily of the resistance (R) component (Table 1, Fig 3B). Potential drop activities Apds1 were visualized as sharp downward spikes of short (1–2 s) duration (Table 3, Fig 2D). Typically, the Apds1 occurred in series of approximately 15 (Fig 2C). The voltage level of successive initial Apds1 in the series gradually dropped to lower values and towards the end of the Apds1 series, the voltage level of the final Apds1 came back to the level similar to Apds1 at the beginning of the series. The series of Apds1 were separated by periods of stylet activities at positive voltage level, usually 5–10 s long (Fig 2C). The predominant electrical component of Apds1 was electromotive force (emf) with a slight addition of R component. Apd1 pattern remained essentially the same after voltage adjustments, but some inversion of the later peaks following the voltage adjustment also occurred (Table 1, Fig 3B). The mean number of Apds1 per minute of AC pathway was over 4 (Table 3). Mean duration of AC2 events per insect were about 24 min long and events occurred more than 8 times during the 8-hour recording, on average (Table 2). Waveform AC2 was detected in 65.1% of the EPG recordings of *A*. *laricis* with AC phase and it constituted 44.5% of total AC time.

**Table 3:** Potential drops during AC phase stylet activities of *Adelges laricis* on *Larix decidua*.

EPG parameters	Apd1	Apd2
	Mean±SE (Min−Max)	Mean±SE (Min−Max)
Number of individuals with potential drops during pathway phase AC^a^	24/43	11/43
Number of potential drops per individual	636.2±232.9 (3.0−422.0)	16.4±8.1 (1.0−94.0)
Mean duration of individual potential drop (s)	1.1±0.1 (0.3−3.2)	9.4±0.8 (5.0−12.6)
Number of potential drops/1 min of path AC^b^	4.19±0.95 (0.01−20.78)	0.15±0.05 (0.01−0.46)
Proportion of Apd1 or Apd2 in total number of Apds (%)	97.9	2.1

Values are means±SE. Lowest and highest values in brackets (Min−Max).

^a^ Number of individuals with pathway phase AC (n = 43).

^b^ Number of Apd1/1 min path AC; number of Apd2/1 min path AC.

Waveform AC3 was also a complex pattern composed of stylet activities at positive voltage levels and potential drops to negative level (Apd2) (Fig 2A). However, unlike in AC2, two types of potential drops occurred: short Apd1 described earlier in waveform AC2 and standard ‘aphid-like’ potential drops Apd2 (Fig 2D–2F). The component of AC3 at positive voltage level consisted of waves of low amplitude and irregular frequency, whose electrical origin was based mainly on resistance (R) component (Table 1). Apds1 occurred frequently but not in a serial regularity as described in AC2 (Fig 2A). Usually, a number of Apd1 preceded Apd2 (Fig 2F). Apds2 were identified as standard ‘aphid-like’ potential drops due to their evident similarity to pds known from pathway (C) activities in aphids. A typical Apd2 was about 10s long and consisted of three phases I, II, and III (Table 3, Fig 2E).

Apd2 phase I included the descending edge of the Apd2 to the negative level. Phase II formed the period of a maintained low potential level and was subdivided into three sub-phases II-1, II-2, and II-3. Apd2 subphases II-1 and II-2 had positive peaks and sub-phase II-3 was shorter and the waves in this sub-phase had lower amplitude. During the phase III the signal returned to the original potential level of pattern AC3 (Fig 2E). On average, less than one Apds2 event occurred per minute of AC pathway (Table 3). Individual AC3 events were shorter and occurred less frequent during the 8-hour recording on average than individual AC2 events. Waveform AC3 was detected in 25.5% of the EPG recordings of *A*. *laricis* that showed pathway phase AC and it constituted 8.0% of total AC time.

Waveforms AC were recorded in more than half studied individuals (Table 2). During the AC phase, the waveforms AC1 and AC2 predominated.

#### Phloem phase: Waveforms AE

**Fig 4:**
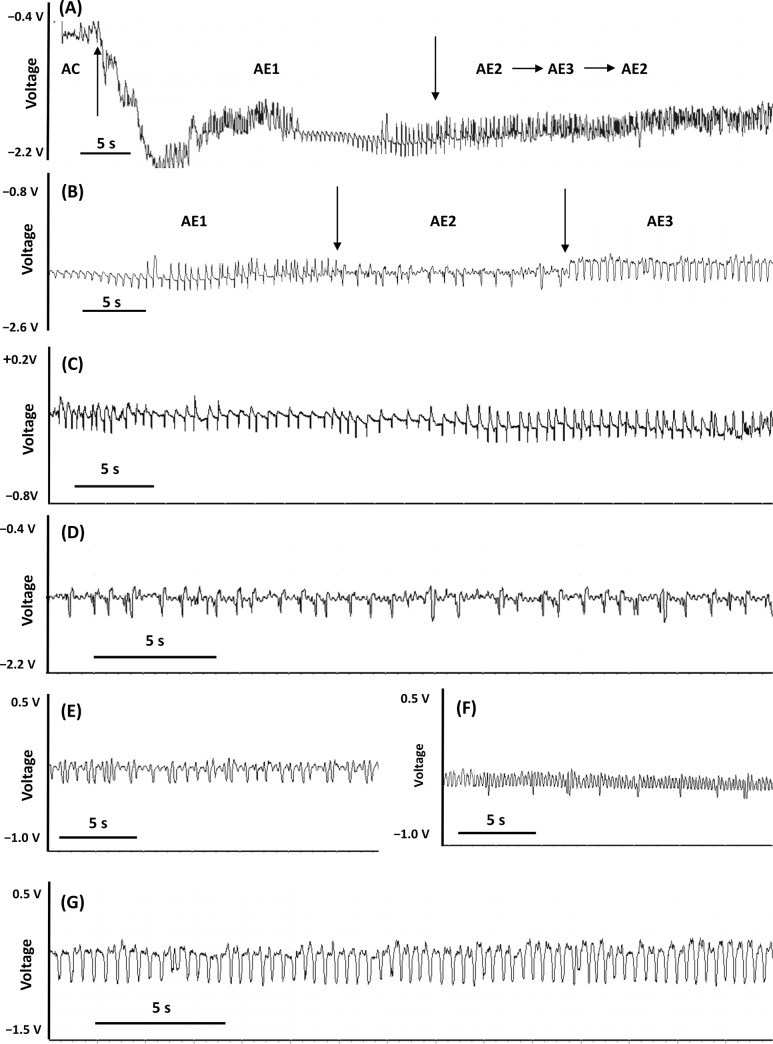
Phloem phase ‘AE’ waveforms generated by *Adelges laricis* during EPG-recorded probing on *Larix decidua*. (A) Transitions from AC to AE1, then AE1 to AE2 and AE2 to AE3; arrows show the transition points; 60 s trace, 2.5V resolution. (B) Compressed view of AE waveforms; arrows show the transition points; 30 s trace, 2.5V resolution. (C) Waveform AE1; 30 s trace, 1V resolution. (D) Waveform AE2; 10 s trace, 2.5V resolution. (E) and (F) Different representations of waveform AE2; 30 s traces, 1V resolution. (G) Waveform AE3; 30 s trace, 1V resolution.

Three variants of AE waveforms were distinguished: AE1, AE2, and AE3. All AE waveforms occurred exclusively at negative voltage level of EPG traces (Fig 4A and 4B).

Waveform AE1 was always the first activity observed after the drop of potential (Fig 4A). AE1 typically had low amplitude upward spikes of regular frequency (up to 4/s) and low voltage level (Table 1, Fig 4B and 4C). The electrical origin of AE1 was electromotive force (emf) (Table 1, Fig 3C). Mean duration of individual AE1 events per insect were about one minute long and events occurred more than 16 times during the 8-hour recording on average. Waveform AE1 was detected in 68.8% of the EPG recordings of *A*. *laricis* that contained AE phase and it constituted 1.2% of total AE time.

Waveform AE2 passed smoothly from waveform AE1 and was composed of negative peaks superimposed on a wave baseline (Fig 4B and 4D–4F). The peaks occurred at a much lower frequency than waves (0.5−1 Hz and 4−5 Hz for peaks and waves, respectively) (Table 1). The peaks had R and the waves had emf as main electrical components (Fig 3D). Mean duration of AE2 events averaged per insect were almost 3 h long, ranging from few minutes to several hours (in some cases even more than 24h –KD personal observation). The AE2 events occurred almost 14 times during the 8-hour recording on average. AE2 was observed in 94.8% of individuals of *A*. *laricis* with AE phase and it constituted 76.6% of total AE time.

Waveform AE3 was mainly preceded by waveform AE2 but periods of AE2 often alternated with AE3 (Fig 4A). AE3 waveform pattern was characterized by a very regular periodicity with peaks and sharp downward spikes, with high relative frequency (up to 7 Hz), and amplitude (no more than 30%) (Table 1, Fig 4G). The shape of AE3 was rather sinusoidal with flattened peaks. The peaks of AE3 usually remained unchanged during voltage adjustments, which suggested electromotive force as main electrical component (Table 1, Fig 3E). However, in some cases, the effects of voltage adjustment caused the inversion of AE3 peaks (Fig 3F). These findings support that both R and emf components were involved in the origin of AE3. Thus the electrical origin of AE3 was of dual nature. Mean duration of AE3 events averaged per insect were about 1,5 h long, ranging from few minutes to several hours. The AE3 events occurred almost 6 times during the 8-hour recording on average. Waveform AE3 was detected in 40.3% of the EPG recordings of *A*. *laricis* that contained AE phase and it constituted 22.3% of total AE time.

Waveforms AE were registered in almost 92% of studied individuals (Table 2). The total duration of waveform AE2 was relatively longer than AE1 and AE3.

#### Xylem phase: Waveform AG

**Fig 5:**
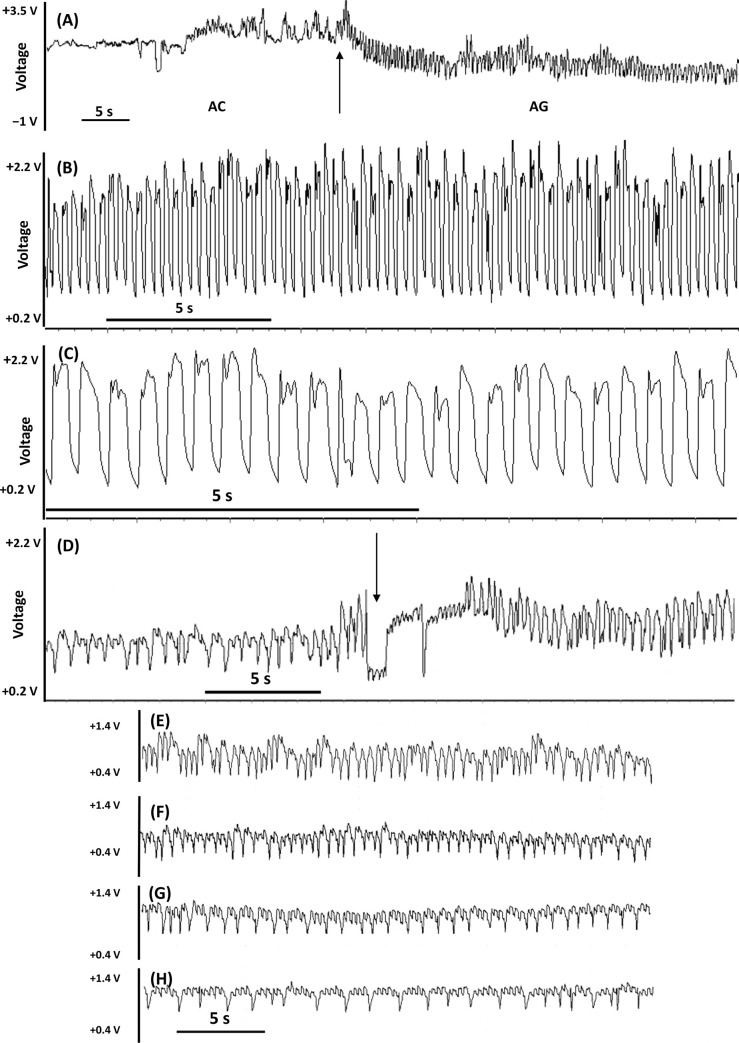
Xylem phase ‘AG’ waveforms generated by *Adelges laricis* during EPG-recorded probing on *Larix decidua*. (A) Transition from AC1 to AG; arrow shows the transition point; 60 s trace, 5V resolution. (B) Waveform AG; 30 s trace, 2.5V resolution. (C) Waveform AG; 10 s trace, 2.5V resolution. (D) Different representations of waveform AG; arrow shows the transition point; 30 s traces, 2.5V resolution. (E)–(H) Different representations of waveform AG; 30 s traces, 1V resolution.

Waveform pattern AG was characterized by the presence of very regular repetition peaks with a low frequency (no more than 2/s) and relatively high amplitude (even to 70%)exclusively at positive voltage level (Table 1, Fig 5A–5C).

Typically, the AG waveform was fairly regular (Fig 5B and 5C), but in a few cases the pattern lost its typical shape but the frequency of peaks remained constant (Fig 5D–5H). The electrical origin of peaks was mainly an electromotive force (emf). However, both R and emf components were involved (Table 1, Fig 3G). The durations of AG periods were variable, ranging from 1 min to several hours. Mean duration of AG events per insect were about 50 min long and occurred almost 5 times during the 8-hour recording on average (Table 2).

### Characterization of EPG waveforms of *A*. *laricis* on sucrose diets

**Fig 6:**
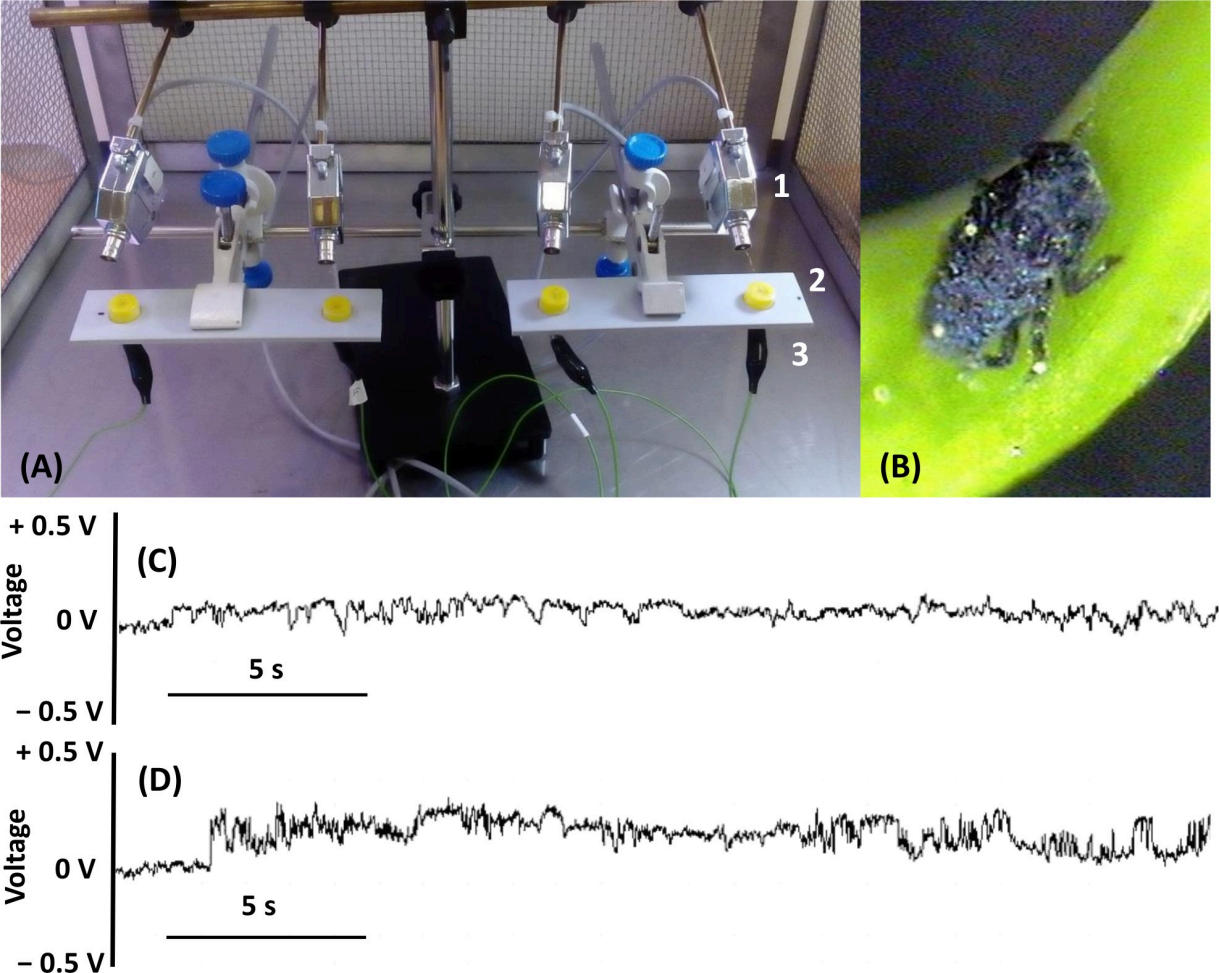
Waveform patterns AC-d generated by *Adelges laricis* on sucrose diets. (A) Equipment for EPG registration on sucrose diets, 1 –aphid electrode, 2 –diet chamber, 3 –diet electrode; (B) the mobile crawler of *A*. *laricis* (C)–(D) representative traces of waveform AC-d; 30 s traces, resolution 1V.

Only one waveform A-d (‘d’ for ‘diet’) was recorded when *A*. *laricis* crawlers were offered a sucrose diet through stretched Parafilm membrane (Fig 6A and 6B). The non-probing phase was followed by stylet penetration (A-d) (Fig 6C and 6D). This pattern was characterized by small amplitude (relative amplitude − 5%) and significant variation in frequency. It consisted of irregular, positive peaks with a large number of spikes. During the waveform A-d, no drops in potential were observed due to the lack of living cells in the diet. The duration of this waveform was very variable, from few seconds to several hours (personal observation KD).

### Stylet penetration activities of *Adelges laricis* on *Larix decidua*

**Fig 7:**
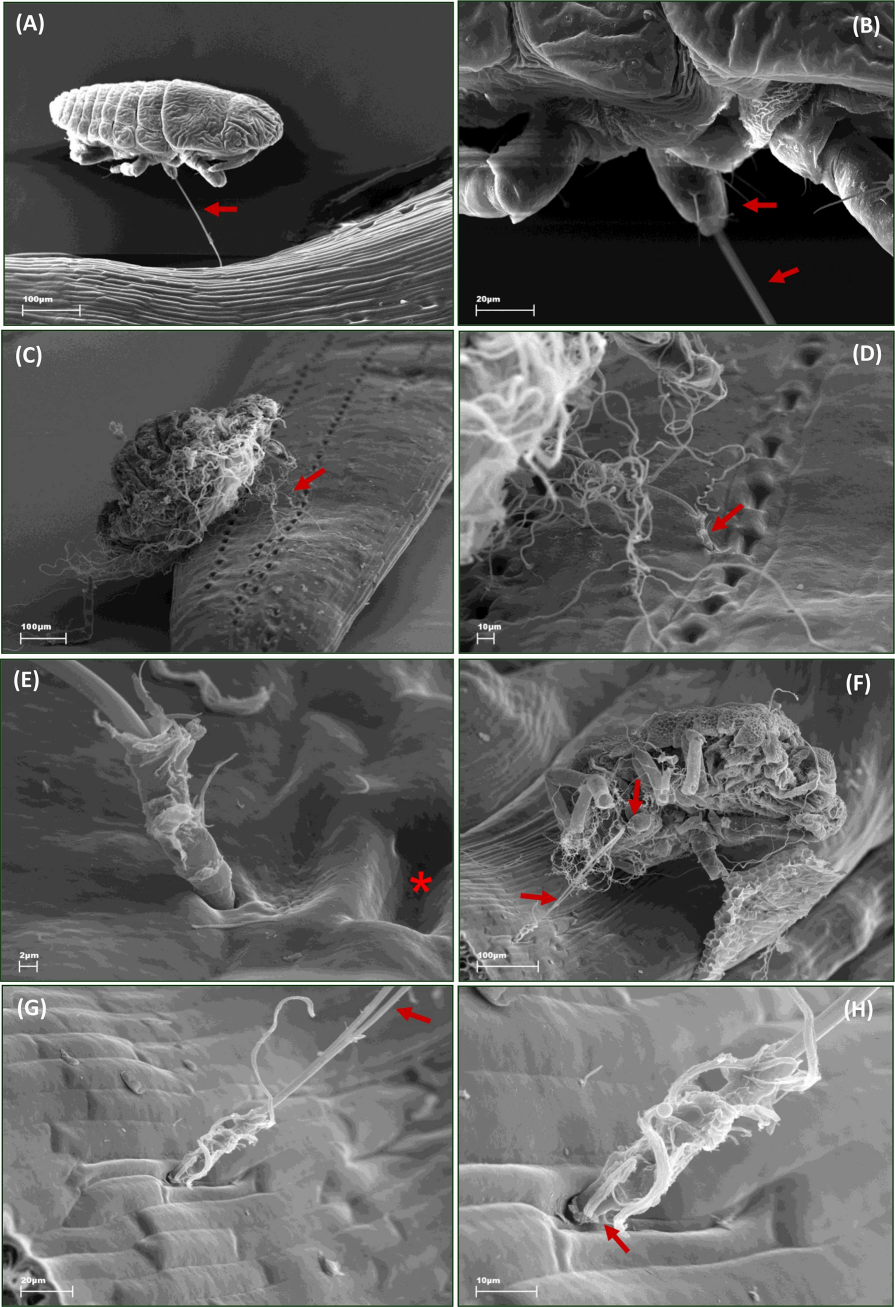
Scanning electron microscope images showing exulis morph of *Adelges laricis* on *Larix decidua* needles. (A) Crawler at the larch needle, lateral view; arrow points at the mouthparts’ stylets. (B) Crawler at the larch needle, ventral view; arrows point at the rostrum and the stylets. (C) Wingless sessile female with most of the wax removed on the larch needle, dorsal view; arrow points at the stylets. (D) Stylets of the female inserted near the stomata of larch needle; arrow points at the stylets. (E) Stylets of the female inserted near a stoma and between epidermal cells of the larch needle; asterisk indicates the stoma. (F) Ventral view at rostrum and stylets of the female; arrow points at the stylets. (G)–(H) Stylets inserted between epidermal cells of the larch needle; different resolutions.

Larch woolly adelgids have long stylets (Fig 7A and 7B) and only the crawlers, the first instar nymphs of exules are active for 1–2 days (personal observation KD). During that period, crawlers search for a suitable site on the larch needles and insert their stylets into the plant tissue (Fig 7A–7C). The stylets are typically inserted intercellularly near the stomata of larch needles and this is true for both the crawlers and the nymphs of later stages and adult females (Fig 7A–7F).

**Fig 8:**
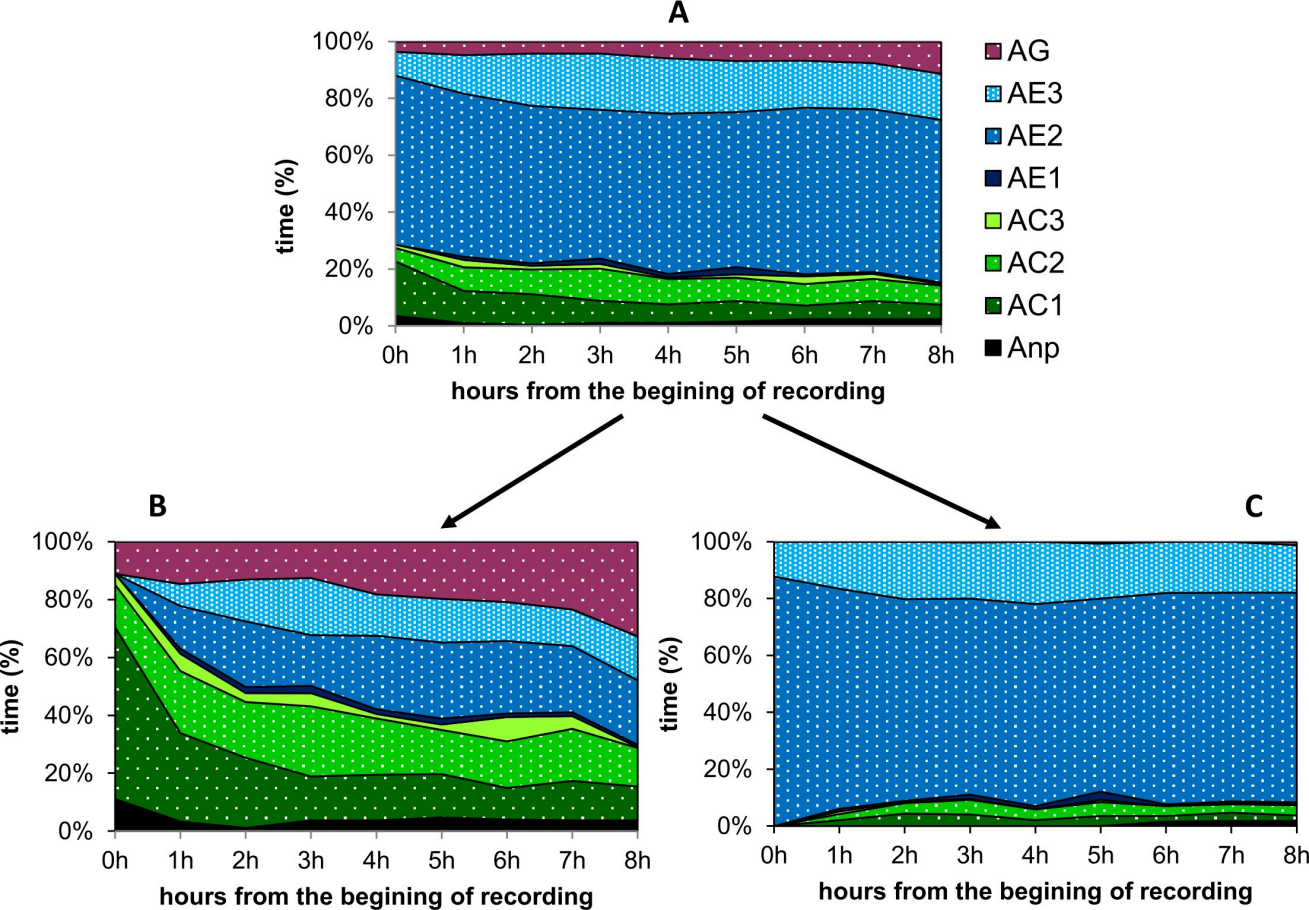
Sequential changes in the probing behavior of *Adelges laricis* on *Larix decidua*. The graphs represent the proportion of time devoted to a given activity at the end of each hour of the 8-hour continuous EPG monitoring. (A) Image based on all replications (n = 84). (B) Image based on recordings that started with either any of AC or AG waveform patterns (n = 24). (C) Image based on recordings that started with any of AE waveform patterns (n = 57). Anp–non-probing. AC1, AC2, AC3 –variants of pathway phase AC. AE1, AE2, AE3 –variants of phloem phase AE. AG–xylem phase.

In the present study, for the reasons stated earlier (see Material and methods), the majority of the studied individuals were wired to the gold wire electrode of the EPG system while their stylets already had been inserted in plant tissues and probing activities were in progress. Only three EPG recordings (3.6%) commenced with non-probing phase (np). All recorded sessile nymphs and adult females that had their stylets inserted at the wiring time (94% of all recorded adelgids) never withdrew their stylets during the entire 8-h recording period. Each of these recordings contained one probe which has not ended before the termination of the experiment. Therefore, the image of sequential changes in adelgid probing behavior during the 8-hour monitoring depended on the way of analysing data (Fig 8A–8C).

The analysis of the whole population of the recorded adelgids (n = 84) showed that AE-related activities predominated during the whole period of 8-hour monitoring (Fig 8A). In the recordings that commenced with EPG waveforms AC or AG (n = 24), the proportion of these stylet activities occupied over half of the adelgid stylet activities during the 8-hour experiment. AE phase appeared during the first and second hour and continued until the end of the experiment. In total, AE-related behavior (waveforms AE1, AE2 and AE3) constituted approximately 35−40% of all probing activities by the end of the monitoring period (Fig 8B). The beginning of recording with one of the AC waveforms or waveform AG occurred in 25% and 4% recordings, respectively. The recordings that started while an adelgid generated AE2 or AE3 waveforms (59.5% and 8.3% of recorded individuals, respectively) were the most common (in 57 of 84 recorded individuals). In this group of recorded adelgids, AE-related activities comprised 90% of all stylet activities in larch tissues during the whole period of monitoring (Fig 8C). The AC-related activities (mainly waveforms AC1 and AC2) occurred marginally and comprised 5% to 9% of all activities of *A*. *laricis* during the whole 8-h recording period (Fig 8C).

**Fig 9:**
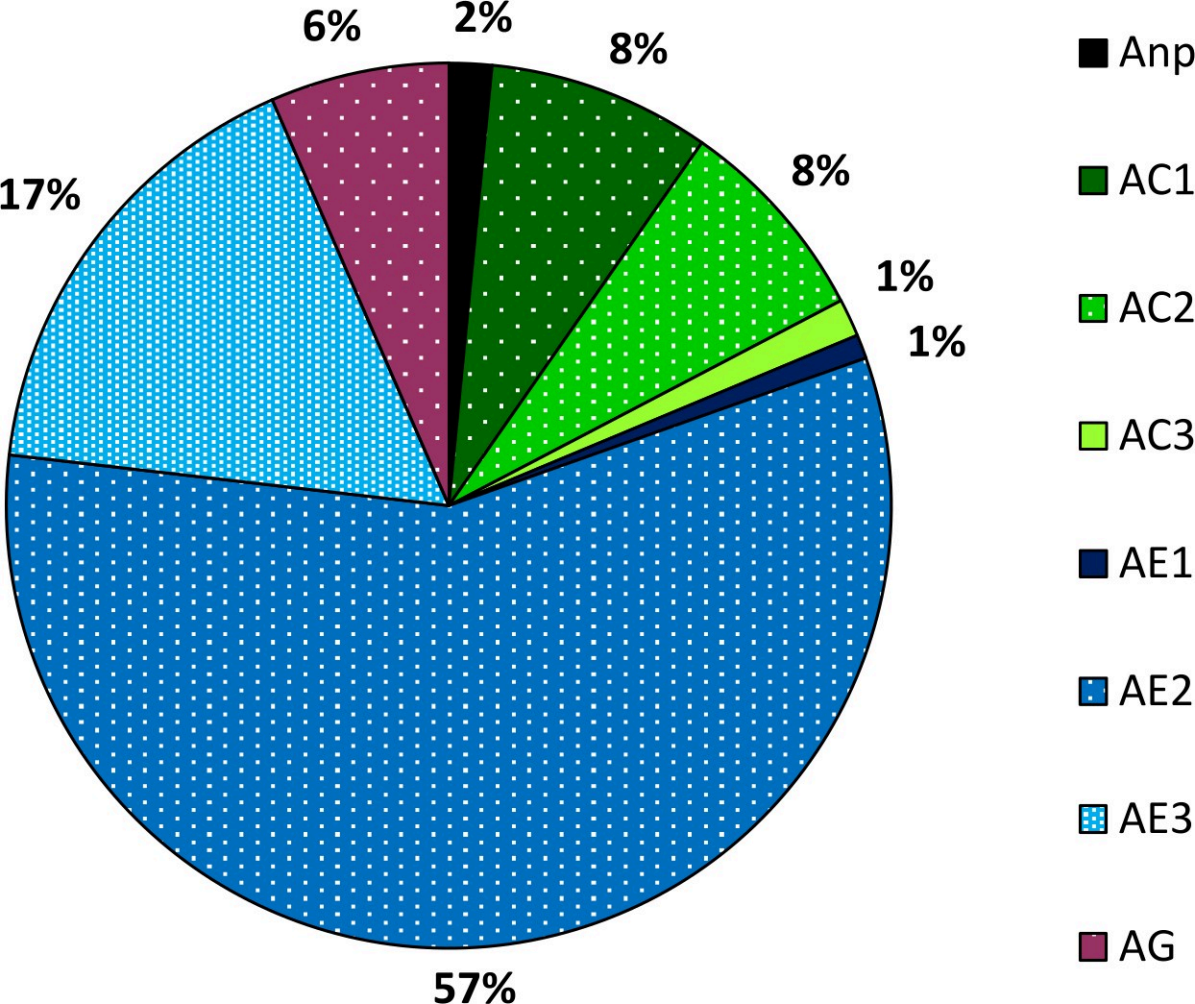
Proportion (%) of various non-probing and probing activities during the 8 h EPG-recordings of *Adelges laricis* on *Larix decidua*. Anp–non-probing. AC1, AC2, AC3 –variants of pathway phase AC. AE1, AE2, AE3 –variants of phloem phase AE. AG–xylem phase.

Of the total 672 h of EPG recordings, *A*. *laricis* spent the least of the time on probing activities related with waveforms AC3 and AE1 and the most of the time on probing activities related with waveform AE2 (Fig 9).

**Fig 10:**
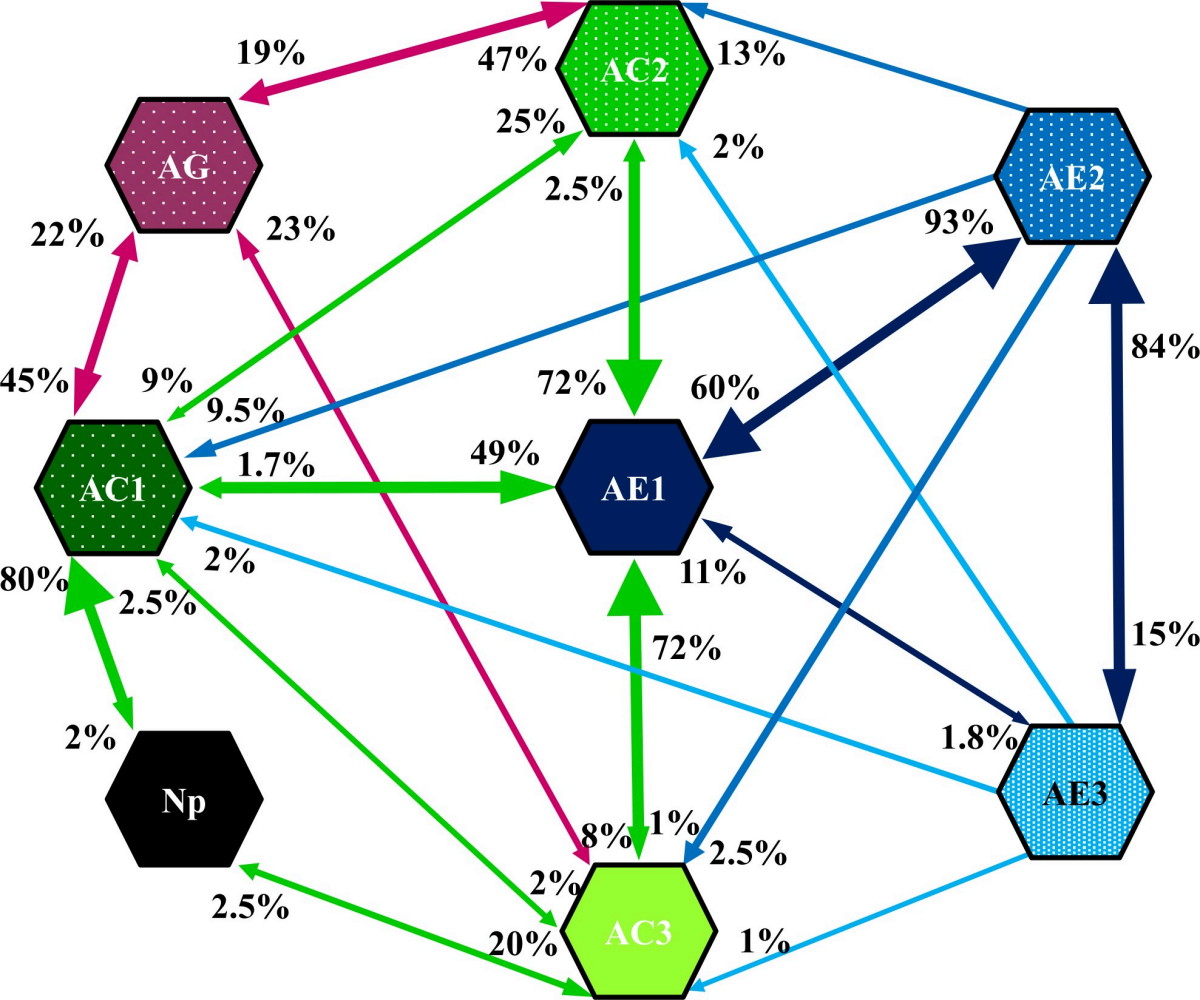
Kinetogram of behavior during stylet penetration of *Adelges laricis* on *Larix decidua*. The values near the arrowheads indicate the occurrence (%) of transitions from one waveform to the next that occurred in all (n = 84) EPG recordings. Anp–non-probing (black labels). AC1, AC2, AC3 –variants of pathway phase AC (green labels). AE1, AE2, AE3 –variants of phloem phase AE (blue labels). AG–xylem phase (purple label).

The non-probing activities were always followed by AC-related stylet probing (waveform AC1 or AC3) (Fig 10).

Waveform AC1 was more often (25%) turned into waveform AC2 with short potential drops (Apd1) than (2%) into waveform AC3 with both short and standard ‘aphid-like’ potential drops (Apd1 and Apd2). There was not a single case of the transfer of AC2 pattern to AC3 and *vice versa*. The activities AC1, AC2, and AC3 were often switched to waveform AG and this occurred in 22%, 19%, and 23% occasions, respectively. The AC1, AC2, and AC3 activities turned into waveform AE1 in 49%, 72%, and 72% of the transitions, respectively. Waveform AE1 was almost always followed by pattern AE2 (93% of the cases), and the AE2 pattern was sometimes followed by pattern AE3 (15% of the cases) or returned back to waveform AE1 (60% of the cases). The pattern AE3 turned into pattern AE2 and AE1 in 84% and 11% of the transitions, respectively. Therefore, the most likely sequence of events during a probe by *A*. *laricis* was stylet penetration with waveforms AC1, AC2 or AC3, followed by AE1 and then AE2 or AE3. Interestingly, the transition from AC2 or AC3 to waveform AE1 was just as likely and moreover, no transitions between these two waveforms of pathway phase AC were observed. Among waveforms of phloem phase AE, the most likely transition was from AE1 to AE2, then from AE3 to AE2 and only then from AE2 to AE1(Fig 10).

## Discussion

### Interpretation of EPG waveforms generated by *Adelges laricis* on *Larix decidua*

Generally, three main categories of EPG waveforms generated by *A*. *laricis* during probing on *L*. *decidua* were distinguished: (1) three variants of waveforms AC that included signals mainly at positive voltage levels with short drops in potential, which suggested mainly extracellular and occasionally intracellular stylet positions, respectively [36,67], (2) one variant of waveform AG, which occurred entirely at positive voltage level that is typical of extracellular stylet activity [75], and (3) three variants of waveform patterns AE that occurred exclusively at negative voltage levels, which indicated the intracellular position of the stylets.

All variants of waveforms AC resembled the stylet pathway activities in plant tissues typical for aphids, during which the stylets penetrate mainly non-vascular plant tissues in search of phloem or xylem vessels [37,43,71,76–80]. In aphids, the pathway activity is rich in short intracellular punctures in otherwise extracellular stylet penetration. Those punctures (observable as potential drops from the positive voltage level of the EPG recorded pathway signal) represent epidermal, mesophyll, and parenchyma cells punctures associated with the uptake of small samples of cell contents and are involved in host plant selection process [79,81]. In *A*. *laricis*, the variations in appearance of pathway AC differed in the frequency and morphology of the potential drops. The waveform AC1 lacked potential drops and was always the pattern that occurred at the start of the probe. The voltages levels, shapes, frequencies and amplitudes of AC1 waves showed resemblance to *A*. *laricis*-derived EPG AC-d waveforms on sucrose diets.

The AC-d waveform appeared soon after labial contact with the parafilm membrane, which suggests that this waveform corresponds to pathway phase and probably represents secretion of salivary sheath and intercellular stylet pathway in any tissue. This analogy between pathway-associated waveforms in plants and waveforms generated during probing in artificial diets has also been reported in aphids [37,42,46,82].

The light microscopy studies demonstrated the evidence of salivary secretions as the indication of *A*. *laricis*, *A*. *tsugae*, and *A*. *abietis* probing, similar to those produced by aphids in plants [16,27,70]. The adelgids seem to probe plant tissues with their stylet bundle sometimes pulling back and then trying a different path within plant tissues without complete withdrawal, thus leaving multibranched salivary tracks in the plant tissue [21,28]. Such multibranched salivary tracks are common in aphid probing [70]. Interestingly, the woolly poplar aphid, *Phloemyzus passerinii* (Signoret), a monoecious aphid with short stylets that probes on poplar trunks, penetrated its stylets into the plant tissues following a straight, unbranched pathway to reach the cortical parenchyma. Compared to EPGs for phloem sap-ingesting aphids, the stylet pathway of *P*. *passerinii* was extracellular but without typical (for aphids) pds [43]. Likewise, pds were not observed during C waveforms of *Phylloxera coccinea* [61] and grape phylloxera (*Daktulosphaira vitifoliae* Fitch) [62] that ingest from parenchyma cell contents. Also in the family Adelgidae, waveforms M at positive voltage level and without potential drops, comparable to the waveform AC1 in *A*. *laricis*, were reported in *Pineus boerneri* Annand on *Pinus taeda* L. [63]. Waveform AC1 occurs not only at the beginning of a probe but also alternates with other pathway phases AC2 and AC3, and quite frequently precedes AG (the putative xylem phase) or AE1 (the putative phloem salivation phase) waveforms, which further supports the explanation that AC1 represents intercellular progressive or regressive movements of stylets within plant tissues as well as the formation of the salivary sheath.

Waveform AC2 was distinguished because it contained a multitude of short potential drops Apd1 arranged in serial patterns and separated by very short intervals of waveforms at positive voltage level. In EPG recordings, the structure of potential drops shows that the insertion of the stylets into a living cell causes minimal and repairable damage to the integrity of the cell membrane [83–86]. The short potential drops Apd1 frequently observed in EPGs of *A*. *laricis* are absent in aphids [66,68]. However, similar to Apd1, short pds were observed in mealybugs *Planococcus citri* (Risso) (Hemiptera: Pseudococcidae) [52] and in common brown leafhopper *Orosius orientalis* (Matsumura) (Hemiptera: Cicadellidae) [59]. In *P*. *citri*, short potential drops occurred during the ‘pre-pd’ phases within pathway activity just before a standard potential drop [52]. The ‘Opds’ in *O*. *orientalis’* begin with a sudden steep fall in voltage into the intracellular level and end in a slow gradual rise in voltage onto the extracellular level, which probably visualizes the damage to the cell membrane by the relatively large stylets of *O*. *orientalis* [59]. In contrast, in the Apd1 generated by *A*. *laricis*, the fall and rise in voltage are equally steep, which indicates that the adelgid stylets do not damage cells during insertion and withdrawal, allowing the recovery of plant cell potential [36,67]. Interestingly, as stated earlier, the short potential drops Apd1 occur in series. Within a series, each Apd1 is separated from the next Apd1 by a very short, 2–3 s interval of extracellular activities, which suggests that a given cell is repeatedly tapped by adelgid stylets on their route.

Waveform AC3 was distinguished because it included short potentail drops (Apd1) and standard aphid-like potential drops (Apd2) within the periods of extracellular pathway. Typical C pathway stylet activities in aphids include 5–10 s potential drops [35,67,77–79]. The Apd2 in AC3 pathway in *A*. *laricis* on larch needles were less frequent than in aphids, but their mean duration was similar to aphid pds. Similarly, as in aphids, Apd2 consisted of three phases I, II, and III and Apd2 subphases II-1 and II-2 with positive peaks, which were similar to respective sub-phases of typical pds in aphids. Also, sub-phase II-3 was shorter and the waves in this sub-phase had lower amplitude than for aphids [61,78,86–92]. In 72% of recorded cases, the waveform AC3 preceded the first observed activities AE1 in the phloem tissue. The potential drops in AC3 are at a similar voltage level as the drop in potential at the beginning of AE1. Therefore, it is very likely that the standard Apd2 potential drops in *A*. *laricis* waveform AC3 reflect short punctures into sieve elements, which is also the case in aphids [88,89,93].

Previously published light microscopy studies suggest that adelgids penetrate plant tissues mainly intracellularly from the insertion site to the ingesting site in the plant [16,21,27]. Indeed, the photographs of microscopic preparations show adelgid stylets crossing the parenchyma cells [16,27]. However, the results of the present study point to the contrary. The structure of potential drops Apd1, specifically the steep rise in potential at the end of the cell puncture event, indicates that cells are probed without permanently damaging the integrity of plasmalemma. The images of adelgid stylets destroying the cells on their route should rather have been interpreted as stylets laying over the layer of parenchyma cells. Similar misinterpretations of light microscopy studies were common in aphid research until electron microscopy studies were published that demonstrated that aphid stylets leave the punctured cells intact in most cases [70,86]. Certainly, the damage of cells by the probing stylets cannot be excluded but it seems that the typical AC pathway of adelgid stylets in non-vascular tissues is intercellular with brief cell punctures.

All waveform AG traces generated by *A*. *laricis* on *L*. *decidua* occurred at positive voltage level and were morphologically similar to the waveform G observed in aphids [37,71,94], mealybugs (Hemiptera, Pseudococcidae) [51,52], whiteflies [95], spittlebugs [96], and psyllids [49,50]. The difference between AG and G was the lower frequency (0.5–2 Hz in *A*. *laricis* and 4–6 Hz in aphids) and the relatively higher emf amplitude of peaks in AG than in G (8–32% relative amplitude and 0–60%) [76,88,97]. G-like waveforms are generated also by aphids and mealybugs during penetration of the artificial diets [37,42,46,52,82]. Hydrostatic pressure of the sap in xylem vessels is generally negative [98,99], therefore waveforms G have been correlated with active ingestion of xylem sap or artificial diet and associated with the activity of the cibarial pump [46,75,76,80,100,101]. The variability in the shape of the AG waveforms observed in our study might have resulted from the quality of the electrical signal [101]. No G-like waveforms at positive voltage level have been described in the phyloxerids *P*. *coccinea*, *D*. *vitifoliae* and the aphid *P*. *passerinii* that ingest from parenchyma cells [43,61,62]. We therefore deduce that the waveform AG in *A*. *laricis* represents an active ingestion of xylem sap.

All waveform AE traces generated by *A*. *laricis* on *L*. *decidua* occurred at negative voltage level. Two variants of AE labelled AE1 and AE2 were morphologically similar to the waveforms E1 and E2 observed in aphids, respectively. However, waveform labeled here AE3 has never been reported from Hemiptera.

AE1 was always the first waveform after AC pathway. It occurred after a drop in voltage of similar magnitude to the last potential drop of the preceding AC and remained at a negative voltage level for the entire duration. The AE1 waveform remained the same after voltage adjustments, so its main electrical component was an electromotive force (emf). AE1 has very similar peaks as the E1 waveform reported for aphids [36,39,76,94,97]. In aphids, E1 is associated with the excretion of saliva into the sieve elements caused by the activity of the salivary pump [35,39,80,100]. The salivary pump action of injecting saliva into the punctured cells was also visualized by the E1-like waveforms: G1 and s-Icp-I in *P*. *coccinea* and *P*. *passerinii*, respectively [43,61]. The structure of the salivary pump including the muscles, ducts, and sensillary pores in all adelgid species is identical to the salivary pump in all Aphididae [12]. Varty [14] and Ponsen [12] considered that adelgids probably produce two types of saliva, one that causes the formation of the salivary sheath to protect the stylet bundle, and the other that contains a hypothetical enzymatic substance to liquefy the parenchyma cells of their host plants. Limbu [22] suggests that hemlock woolly adelgid, which ingests from xylem ray parenchyma cells, may inject saliva into the tree to extraorally digest the nutrients before the insect sucks them back up, and in so doing causes a tree-wide defensive response. AE1 is generated after the stylets enter a living cell and the signal remains at the negative voltage level for the whole duration of AE1. Moreover, AE1 is usually followed by AE2 or AE3 waveform that represent the putative ingestion phases. We infer that AE1 can be associated with salivation into sieve elements, as reported for E1 in aphids [85,94].

The AE2 pattern smoothly passes from waveform AE1. The transition waveforms between AE1 and AE2 are similar to those known from aphids [102]. AE2 originates and remains at negative voltage level for the whole duration. The morphology and frequency of peaks and waves of AE2 are similar to those reported for E2 in aphids [39,71,76,86,97]. In aphids, E2 was associated with the passive ingestion of phloem sap and concurrent continuous secretion of saliva [39,80,94]. Also, it was established that only during E2 waveforms, honeydew was excreted by aphids [100]. Ponsen [12] reported that adelgids insert their stylets into the needles of their host plant and subsequently excrete honeydew. Cardoso [63] described the intracellular waveform P in *P*. *boreani* on *Pinus taeda* L. Certain fragments of waveform P resemble the waveform E2 in aphids and AE2 in *A*. *laricis* EPG traces. *P*. *boreani* displayed the pattern P for 22.3 h on average and the excretion of honeydew was observed during this period, which indicated possible phloem sap ingesting [63]. The duration of AE2 waveform was usually very long, from several minutes to several hours (even more than 24h –personal observation KD). It is likely that *A*. *laricis* on its secondary host *L*. *decidua* shows sustained passive fluid intake from the phloem, which is visualized in the AE2 pattern.

The waveform AE3 was usually preceded by waveform AE2 or less often by waveform AE1 and always occurred at negative voltage level which indicated the intracellular position of the stylets. Waveform AE3 lasted from several minutes to several hours and was distinguished because of the difference in morphology and frequency of peaks between AE3 and AE1 and AE2. Briefly, AE3 resembled the AG waveform pattern but it occurred at different voltage level, AE3 –intracellular and AG–extracellular. At the same time, AG resembles the G waveform known from aphids, as commented earlier. The frequency of peaks in AE3 was slightly lower than in G waveform for aphids [76,97]. The peaks in aphid G pattern were correlated with the activity of the cibarial pump and the active ingestion of fluids from xylem and artificial diets [76,80,100,101]. Angiosperm and gymnosperm trees have a fundamentally different phloem anatomy with respect to cell size, shape and connectivity. The sieve elements (SEs) of gymnosperms are generally longer and thinner than in angiosperms and devoid of sieve plates. The wide open sieve pores in the angiosperm sieve plate contrast with the plasmodesmata-like cell connections in the tapering end walls of gymnosperm SEs. Pores in these sieve areas are filled with lamellar structures (tubular-vesicular ER) [103,104]. The anatomical differences led to speculation whether gymnosperms might use a different mechanism for whole-plant phloem transport [105,106]. Analysis of the model variables clearly identified SE anatomy. Also, a meta-analysis of the experimental data showed that hydraulic resistance is significantly higher and phloem transport speed is slower in gymnosperm trees than that in angiosperm trees (average 22 cm h^-1^ and 56 cm h^-1^, respectively) [104]. The occurrence of G-like waveforms at intracellular level has been reported in aphids and phylloxerids [43,61]. During the ingesting on parenchyma cell contents, *P*. *passerinii* and *P*. *coccinea* generate EPG waveforms s-Icp-II and G2, respectively, which resemble the G pattern of Aphididae but occur at the negative voltage level, which suggests an active mode of sap ingestion [43,61].

The fact that the E2 waveform that represents passive mode of sap ingestion was never recorded in aphids on the artificial diets was presumably due to the lack of sufficient hydrostatic pressure, which forced the insect to ingest food actively, using the cibarial pump. A waveform similar to G was associated with an active mode of ingestion of the liquid diet [46,82,100]. Active ingestion of phloem sap may be necessary due to special structure and physiology of phloem in gymnosperms. Based on this information and the analogy between AE3 and AG and G waveforms and their meaning, we hypothesize that the waveform AE3 represents active phloem sap ingestion. However, this hypothesis is open for discussion and needs confirmation in further studies. That said, waveform AE3 has been described for the first time in Hemiptera. We propose that the unique character of waveform AE3 derives from the special nature of adelgid-gymnosperm plant association. AE3 demonstrates the adjustment of adelgid stylet penetration activities to the individuality of phloem structure in conifer plants.

### Proposed probing behavior of *Adelges laricis* on *Larix decidua*

*Adelges laricis* alternates between spruce and larch to complete its life cycle. On spruce, which is considered the primary host, sexual reproduction and gall formation occurs [13]. Larch, the secondary host, supports several parthenogenetic generations of *A*. *laricis*. All morphs that develop on larch exploit the needles of the tree, but without stimulating gall formation [20]. *A*. *laricis* on the primary host is a cortical parenchyma ingester who can modify the cortical cells and cause them to generate structures similar to those of the phloem, which allows easy transport of the solutes from the vascular bundles of the young shoot [27]. The probing and cell puncturing by the first larval stage of the fundatrix induces cell dedifferentiation and activates tissue hyperplasy and hypertrophy. In this transformed tissue, starch and secondary metabolites accumulate. Cell autolysis occurs and the parenchyma cells around the stylet tips become nearly empty of their content, which indicates the ingestion of the accumulated cell content [27]. On the secondary hosts of adelgids, the situation is more complex. Many studies imply that on secondary hosts, the Adelgidae exploit both parenchyma cells and phloem sieve elements as their food supply. On fir *Abies alba* Mill., *Adelges nüsslini* (Börner) infests the needles and the old and young stems [14]. When *A*. *nüsslini* settles on the needles, ingestion takes place from the conductive tissues beyond the endodermis. On the main stem of an old tree, the sistentes, i.e. the exulis diapausing morphs of *A*. *nüsslini* ingest from the storage parenchyma and their stylets can penetrate to the cambium, so that the xylem and phloem differentiate normally. In the young twigs, the stylets reach the tile zone of the cambium and the conducting tissues. The twig shows hyperplasia and hypertrophy of abnormal parenchyma in place of vascular tissue [14]. The deterioration of phloem was described also in *Pinus strobus* L. shoots infested by the exulis, i.e., the parthenogenetic morphs of *Pineus pinifoliae* (Fitch) [31]. Phloem deterioration is characterized by hypertrophied young sieve elements and cellular breakdown in the phloem region, destruction of vascular cambium, and ultimately, in the formation of large structureless cavities surrounding the xylem. Internal deterioration is evidenced externally by needle discoloration followed by twig drooping [31].

The stylets of the exulis of *P*. *pinifoliae* follow an intercellular course through the cortex of *P*. *strobus* and terminate in the young sieve cells of the phloem [31]. The mouthparts of the sedentary apterous forms of *Pineus strobi* (Hartig) on *P*. *strobus* extend at least into the outer tissues of the phloem [30]. The apterous and winged progredientes, i.e., the non-diapausing parthenogenetic morphs of *Adelges cooleyi* (Gillette) insert their stylets into the phloem cells when probing on newly emerging needles of Douglas fir (*Pseudotsuga menziesii* (Mirbel)) [33]. The pattern of stylet activities by of *A*. *laricis* on the secondary host has never been explored. We have demonstrated that *A*. *laricis* may ingest sap form both xylem and phloem vessels. We examined the probing behavior of 84 individuals and only 25% the monitored adelgids showed periods of waveform AG that represents the ingestion of xylem sap. The mean duration of an AG period was less that one hour and the AG pattern was followed usually by pathway activities AC1 or AC2. Thus, we consider it unlikely that *A*. *laricis* uses xylem sap as a main source of nutrients. The Adelgidae may only occasionally ingest xylem sap, which might be an important source of water for avoiding dehydration, as has been shown for aphids [75]. In contrast, waveforms AE, mainly the variants AE2 and AE3, were recorded in 92% of individuals. AE3 alternated with AE2 but AE3 occurred less frequently than AE2. Nevertheless, the periods of AE2+AE3 lasted for many hours without interruption. In our opinion, the phloem vessels, namely the sieve elements are the basic source of food for *A*. *laricis* on *L*. *decidua*.

In summary, this study provides the first detailed and original information about various aspects of probing, including and ingestion behaviors of *A*. *laricis* on secondary host *L*. *decidua*. The search for the ingestion site is initiated by the only mobile morphs, the crawlers that hatch from the eggs and have longer legs and antennae than the sessile advanced nymphs and females that follow. The selection of ingestion sites does not occur accidentally, a typical series of behavioral events eventually leads to sustained phloem sap ingestion. The stylets are typically inserted intercellularly near the stomata of the larch needles. Probing consists of extra- and intracellular stylet activities and the main extracellular activity is pathway that includes cell punctures. The similarities in adelgid EPG AC waveform characteristics to aphid-generated EPG C waveforms show that it is most likely that waveforms AC visualize the stylet penetration in non-vascular tissues. Waveforms AC1 or AC2 that predominate in pathway activities probably represent the secretion of salivary sheath and intercellular stylet pathway without (AC1) or with (AC2) intracellular punctures. Waveform AC3 probably represents stylet pathway with intracellular short and standard punctures close to or within the vascular bundle. On secondary hosts, *A*. *laricis* ingests phloem sap that may be either passively or actively. The presence of waveforms AG and AE3 during probing on larch needles indicates that *A*.*laricis* may be able to actively ingest fluids directly from vascular tissues and that these patterns may be associated with the rhythmic activity of cibarial muscles when ingesting fluid in the form of xylem or phloem sap.

Generally, our results allowed us to establish or infer correlations of *A*.*laricis* waveforms with the stylet tip position in specific plant tissues (inter- and intracellular). However, the correlation of waveforms with stylet probing activities (e.g. saliva secretion, passive or active ingestion) remains hypothetical. Moreover, because more than one waveform is associated with the same tissue (AC1, AC2 and AC3 were all related to the non-vascular tissue, and AE1, AE2 and AE3 to the phloem), the differences among waveforms should be further investigated. Such studies would benefit from a histological approach to the plant and insect combined with laser stylectomy.

## Supporting information

S1 File Frequency and duration of individual EPG-recorded waveforms generated by *Adelges laricis* during probing on *Larix decidua*. d–duration. N–number. Ind-per individual.
(XLSX)

S2 File Potential drops during AC phase stylet activities of *Adelges laricis* on *Larix decidua*. d–duration. N–number. Ind-per individual.
(XLSX)

S3 File Proportion (%) of various non-probing and probing activities during the 8 h EPG-recordings of *Adelges laricis* on *Larix decidua*. (XLSX)

S4 File Sequential changes in the probing behavior of *Adelges laricis* on *Larix decidua*. (XLSX)

S5 File Percentage (%) of transitions from one waveform to the next that occurred in all (n = 84) EPG recordings. (XLSX)

S6 File Relative amplitude (%)a (min-max). (XLSX)
